# Supplementary material for: Sterol 14α-Demethylase Ligand-Binding Pocket-Mediated Acquired and Intrinsic Azole Resistance in Fungal Pathogens
Source: J Fungi (Basel). 2020 Dec 22;7(1):1. doi: 10.3390/jof7010001 (PMC7822023; doi:10.3390/jof7010001)
Supplement: Supplementary file 1 [file jof-07-00001-s001.pdf]

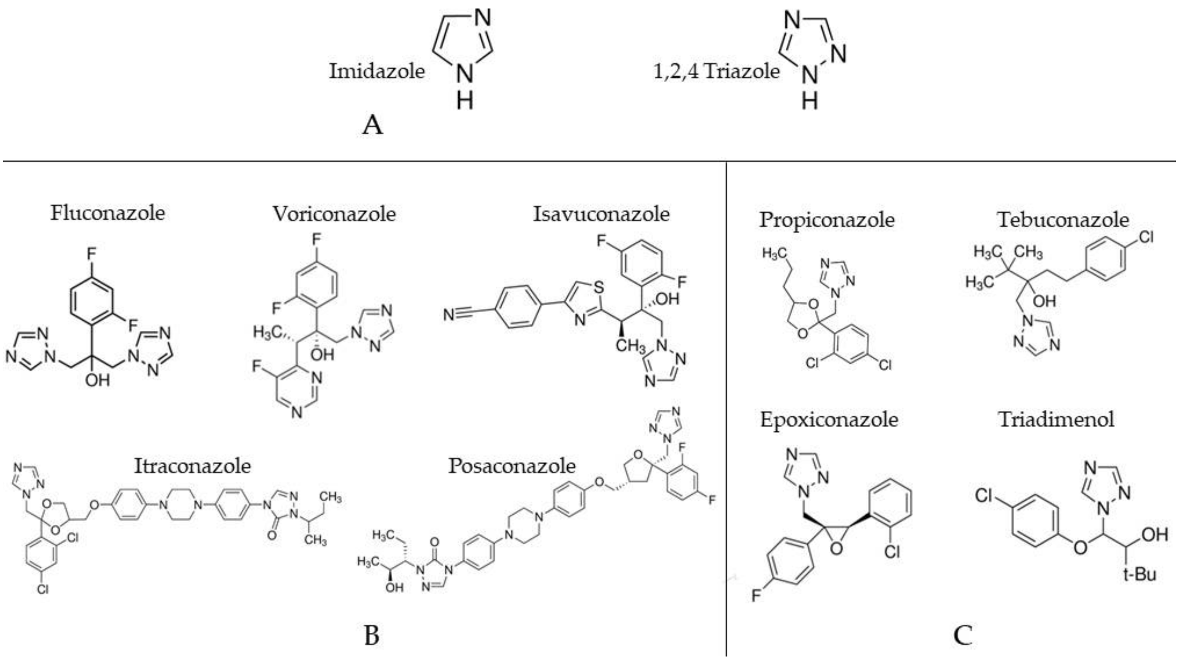

Figure S1: Structures azole drugs and agrochemicals. (A) Imidazole and 1,2,4 triazole substituents. (B) Sterol 14α-demethylase inhibitors used as triazole drugs. (C) Sterol 14α-demethylase inhibitors used as fungicides as agrochemicals. Fluconazole, voriconazole, propiconazole, tebuconazole, epoxiconazole and triadimenol are short-tailed azole drugs. Isavuconazole is a mid-tailed azole drug. Itraconazole and posaconazole are long-tailed azole drugs. Structures were modified from https://www.sigmaaldrich.com, 27.10.2020

Table S1: Sterol 14α-demethylase amino acid substitutions and their phenotypic effects.

Genus	Species	Type of Isolate	AA change	Quality wt	Qualitiy change	2nd Mutation	TR	MICs (µg/mL)							Number strains	Reference
								FLC	VRC	TDM*	IVU	ITC	PSC	AMB		
Ajellomyces	*capsulatus*	environmental	Y136F	polar	Non polar			n.a.	n.a.	n.a.	n.a.	n.a.	n.a.	n.a.	n.a.	[28]
Aspergillus	*fumigatus*	clinical	Y121F	polar	non polar	T289A	TR46	n.a.	4->16	n.a.	16->16	0.5->16	0.25-1	n.a.	17	[32-35]
Aspergillus	*fumigatus*	environmental	Y121F	polar	non polar	T289A	TR46	n.a.	0.25->16	n.a.	0.125->8	0.25->16	0.06-1	n.a.	112	[136,142-144]
Aspergillus	*fumigatus*	environmental	Y121F	polar	non polar			n.a.	n.a.	n.a.	n.a.	n.a.	n.a.	n.a.	n.a.	[78]
Aspergillus	*fumigatus*	mutants	I301	polar	non polar			20	0.12	n.a.	n.a.	0.06	0.25	0.5	1	[36]
Candida	*albicans*	mutants	Y132F	polar	non polar			8	0.5	n.a.	n.a.	0.25	<0.03	n.a.	1	[16]
Candida	*albicans*	mutants	Y132H	uncharged	+ charge			2	0.25	n.a.	n.a.	0.25	<0.03	n.a.	1	[16]
Candida	*auris*	clinical	Y132F	polar	non polar			64->256	0.094-1.5	n.a.	n.a.	n.a.	n.a.	n.a.	12	[96]
Candida	*auris*	clinical	K143R	one N atom	3 N atoms			>256	2-4	n.a.	n.a.	n.a.	n.a.	n.a.	24	[96]
Candida	*parapsilosis*	clinical	Y132F	polar	non polar			8-256	0.12-2	n.a.	n.a.	n.a.	n.a.	n.a.	79	[27,46]
Candida	*parapsilosis*	clinical	Y132F	polar	non polar	R398I		64-128		n.a.	n.a.	n.a.	n.a.	n.a.	9	[46]
Candida	*tropicalis*	clinical	K143R	one N atom	3 N atoms			>64	8	n.a.	n.a.	1	1	n.a.	1	[124]
Cryptococcus	*neoformans*	mutants	Y145F	polar	non polar			128	2-3	n.a.	n.a.	0.094-0.38	0.125-0.25	n.a.	1	[44]
Cryptococcus	*neoformans*	mutants	Y145F	polar	non polar	P6S, C45G, Q50L, S460T		128	2	n.a.	n.a.	0.38	0.25	n.a.	2	[44]
Mucor	*circinelloides*	n.a.	F129	non polar	-	A291		>64	16->16	n.a.	n.a.	n.a.	n.a.	n.a.	18	[49]
Mucor	*circinelloides*	clinical	F129	non polar	-	A291		64	8->16	n.a.	0.125->16	0.25-16	0.015->16	0.03-0.06	20	[145,146]
Rhizopus	*arrhizus*	n.a.	F129	non polar	-	A291		>64	4-16	n.a.	n.a.	n.a.	n.a.	n.a.	17	[49]
Rhizopus	*arrhizus*	clinical	F129	non polar	-	A291		64->64	4->16	n.a.	0.5-8	0.125-16	0.125->16	0.03-0.125	21	[145,146]
Rhizopus	*microsporus*	n.a.	F129	non polar	-	A291		n.a.	4-16	n.a.	n.a.	n.a.	n.a.	n.a.	13	[49]
Rhizopus	*microsporus*	clinical	F129	non polar	-	A291		n.a.	16->16	n.a.	1-8	n.a.	0.5->16	n.a.	26	[145,146]
Rhizopus	*microsporus*	clinical	F129	non polar	-	A291		64	1-16	n.a.	0.125-4	0.06-16	0.06-4	0.03-1	17	[146]
Scedosporium	*apiospermum*	clinical	Y136F	polar	non polar			n.a.	n.a.	n.a.	n.a.	n.a.	n.a.	n.a.	2	[50]
Blumeria	*graminis*	environmental	Y136F	polar	non polar			n.a.	n.a.	n.a.	n.a.	n.a.	n.a.	n.a.	n.a.	[51,52,119]
Mycosphaerella	*graminicola*	environmental	Y137F	polar	non polar			n.a.	n.a.	14.73	n.a.	n.a.	n.a.	n.a.	4	[52]
Parastagonospora	*nodorum*	environmental	Y144F	polar	non polar			n.a.	n.a.	n.a.	n.a.	n.a.	n.a.	n.a.	n.a.	[54]
Puccinia	*triticina*	environmental	Y134F	polar	non polar			n.a.	n.a.	n.a.	n.a.	n.a.	n.a.	n.a.	n.a.	[55]
Uncinula	*necator*	environmental	Y136F	polar	non polar			n.a.	n.a.	8.2-22.1	n.a.	n.a.	n.a.	n.a.	n.a.	[56,147]

Legend: not available (n.a.), amphotericin B (AMB), fluconazole (FLC), itraconazole (ITC), posaconazole (PCZ), voriconazole (VCZ), isavucoanzole (IVU), tandem repeat in the promotor region of the lanosterol 14 alpha demethylase gene (TR), chemical character of wild type amino acid (quality of wt), amino acid (AA), phenylalanine (F), tyrosine (Y), threonine (T), isoleucine (I), arginine (R), lysine (K).

Table S2: EUCAST Clinical Breakpoints (CBPs) of human pathogenic fungi.

	AMB (µg/mL)	FLC (µg/mL)	ITC (µg/mL)	PCZ (µg/mL)	VCZ (µg/mL)	IVU (µg/mL)
Aspergillus flavus	n.a.	n.a.	1	n.a.	n.a.	2
Aspergillus fumigatus	1	n.a.	1	0.25	1	2
Aspergillus nidulans	n.a.	n.a.	1	n.a.	1	0.25
Aspergillus niger	1	n.a.	n.a.	n.a.	n.a.	n.a.
Aspergillus terreus	n.a.	n.a.	1	0.25	n.a.	1
Candida albicans	1	4	0.06	0.06	0.25	n.a.
Candida tropicalis	1	4	0.125	0.06	0.25	n.a.

Legend

Not available (n.a.), amphotericin B (AMB), fluconazole (FLC), itraconazole (ITC), posaconazole (PCZ), voriconazole (VCZ), isavuconazole (IVU)

Clinical Breakpoints according to EUCAST Antifungal Clinical Breakpoint Table v. 10.0 valid from 2020-02-04

Isolates expressing higher minimal inhibitory concentrations (MIC) than listed in the table above are considered resistant, exhibiting a lower or the exact MIC than given in the table above are considered susceptible.

Table S3: Itraconazole and lanosterol binding plus innate resistance in eukaryotic CYP51s. Residues within 4 Å of itraconazole in *S. cerevisiae* SDM are shown in bold. Residues within 4 A° of lanosterol in the human CYP51 D231A H314A mutant that has high substrate occupancy are highlighted in green (in general, possible hydrophobic interactions). Residues with their main chain nitrogen possibly involved in a water-mediated hydrogen bond network with the hydroxyl of lanosterol (M378, M487) are highlighted in gray while the residue (I379) using its main chain carbonyl to make a direct hydrogen bond with lanosterol is highlighted in gray and in **bold**. Some residues were where alignments and some experimental work indicate possible roles in substrate specificity and/or as causes of innate resistance to azole drugs are highlight in light blue. Selected residues identified as relevant to innate azole resistance representative of molds are illustrated using *A. fumigatus* and for mucormycetes they are illustrated using *R. arrhizus.*

Helix B-loop-Helix C (SRS1)
S. cerevisiae LDM 124 AA**Y**AHLTTPV**F**GKGV**IY**DCP
C. albicans LDM 116 DAYKHLTTPVFGKGVIYDCP
A. fumigatus CYP51A 105 EVYSPLTTPVFGSDVVYDCP
A. fumigatus CYP51B 120 EVYSPLTTPVFGRHVVYDCP
R. arrhizus LDM F1 110 DAYNHMTKHVFGPEVVYDAP
R. arrhizus LDM F5 112 AAYNHMTKYVFGNDIVFDTA
H. sapiens CYP51 129 DVYSRLTTPVFGKGVAYDVP

T. aestivum CYP51	109	EVYR-FNVPTFGPGVVFDVP

Helix C

S. cerevisiae LDM	147	LMEQKKFVK
C. albicans LDM	139	LMEQKKFAK
A. fumigatus CYP51A	128	LMEQKKFIK
A. fumigatus CYP51B	143	LMEQKKFIK
R. arrhizus LDM F1	136	FMEQKKFIK
R. arrhizus LDM F5	136	FMEQKRFIK
H. sapiens CYP51	152	FLEQKKMLK
T. aestivum CYP51	1 31	RQEQFRFFT

F-F”

S. cerevisiae LDM	236	**F**-T**P**IN**F**
C. albicans LDM	228	F-TPINF
A. fumigatus CYP51A	214	F-TPINF
A. fumigatus CYP51B	229	F-APINF
R. arrhizus LDM F1	217	F-KPINF
R. arrhizus LDM F5	217	F-RPINF
H. sapiens CYP51	234	FSHAAWL
T. aestivum CYP51	213	M-LPISV

Helix I (SRS3)

S. cerevisiae LDM	306	NLLI**GV**LM**G**GQH**T**SAAT
C. albicans LDM	299	NLLIGILMGGQHTSAST
A. fumigatus CYP51A	285	HMMITLLMAGQHSSSSI
A. fumigatus CYP51B	299	HMMIALLMAGQHSSSST
R. arrhizus LDM F1	286	GMMIAVLFGGQHTSSTT
R. arrhizus LDM F5	286	GILTAALFGGQHTSSTT
H. sapiens CYP51	303	GMLIGLLLAGQHTSSTT
T. aestivum CYP51	281	GLLIAALFAGQHTSSIT

K-K′ loop

```
S. cerevisiae LDM          379 PLHSLFR
C. albicans LDM       299 PLHSIFR
A. fumigatus CYP51A   363 SIHSIMR
A. fumigatus CYP51B   372 PIHSIIR
R. arrhizus LDM F1         347 PIFQMMR
R. arrhizus LDM F5         347 PIFNMR
H. sapiens CYP51      376 PIMIMMR
T. aestivum CYP51          354 PLIMLLR
```

C-terminal region

```
S. cerevisiae LDM          505 DFTS-MVTL
C. albicans LDM       504 DYSS-MVVL
A. fumigatus CYP51A   490 DYSS-LFSG
A. fumigatus CYP51B   499 DYSS-LFSK
R. arrhizus LDM F1         478 DYTS-MVVV
R. arrhizus LDM F5         476 DYTS-MVVV
H. sapiens CYP51      483 NYTT-MIHT
T. aestivum CYP51          467 NDWNAMVVG
```


142. Sewell, T.R.; Zhang, Y.; Brackin, A.P.; Shelton, J.M.G.; Rhodes, J.; Fisher, M.C. Elevated prevalence of azole-resistant *Aspergillus fumigatus* in urban versus rural environments in the United Kingdom. *Antimicrob. Agents Chemother.* **2019**, *63*, e00548-19.
143. Siopi, M.; Rivero-Menendez, O.; Gkotsis, G.; Panara, A.; Thomaidis, N.S.; Alastruey-Izquierdo, A.; Pournaras, S.; Meletiadis, J. Nationwide surveillance of azole-resistant *Aspergillus fumigatus* environmental isolates in Greece: Detection of pan-azole resistance associated with the TR46/Y121F/T289A *cyp51A* mutation. *J. Antimicrob. Chemother.* **2020**, *75*, 3181–3188.
144. Chen, Y.C.; Kuo, S.F.; Wang, H.C.; Wu, C.J.; Lin, Y.S.; Li, W.S.; Lee, C.H. Azole resistance in *Aspergillus* species in southern Taiwan: An epidemiological surveillance study. *Mycoses* **2019**, *62*, 1174–1181.
145. Arendrup, M.C.; Jensen, R.H.; Meletiadis, J. In vitro activity of isavuconazole and comparators against clinical isolates of the *Mucorales* order. *Antimicrob. Agents Chemother.* **2015**, *59*, 7735–7742.
146. Chowdhary, A.; Kathuria, S.; Singh, P.K.; Sharma, B.; Dolatabadi, S.; Hagen, F.; Meis, J.F. Molecular characterization and in vitro antifungal susceptibility of 80 clinical isolates of Mucormycetes in delhi, india. *Mycoses* **2014**, *57*, 97–107.
147. Cools, H.J.; Mullins, J.G.; Fraaije, B.A.; Parker, J.E.; Kelly, D.E.; Lucas, J.A.; Kelly, S.L. Impact of recently emerged sterol 14alpha-demethylase (*cyp51*) variants of *Mycosphaerella graminicola* on azole fungicide sensitivity. *Appl. Environ. Microbiol.* **2011**, *77*, 3830–3837.
